# Difference in cytological findings of healthy and conjunctivitis/keratoconjunctivitis affected canine eyes between variably experienced evaluators

**DOI:** 10.14202/vetworld.2022.1852-1856

**Published:** 2022-07-28

**Authors:** Inese Berzina, Anastasija Terentjeva, Liga Kovalcuka

**Affiliations:** 1 Preclinical Institute, Faculty of Veterinary Medicine, Latvia University of Life Sciences and Technologies, Jelgava, Latvia; 2 Clinical Institute, Faculty of Veterinary Medicine, Latvia University of Life Sciences and Technologies, Jelgava, Latvia

**Keywords:** conjunctival cytology, microscopy experience, neutrophil morphology

## Abstract

**Background and Aim:**

Cytology investigations are a frequent part of ophthalmological examination. We aimed to assess whether the cytological findings of healthy and conjunctivitis/keratoconjunctivitis samples differed based on the evaluator’s experience.

**Materials and Methods:**

A study evaluated healthy eyes (n = 40) and eyes affected with keratoconjunctivitis and/or conjunctivitis (n = 28) in dogs. An ophthalmological examination was performed before sampling the eyes using a sterile cotton swab. An evaluator with theoretical experience and one with undergone clinical pathology residency training performed cytology blinded to the clinical findings.

**Results:**

In the healthy eyes group, the agreement between the evaluators for cellularity was nonexistent, while that for cell preservation and mucus content was fair. In the affected eyes group, the agreement for cellularity and mucus content was moderate, while that for cell preservation was fair. The inadequate sample rate differed significantly between the two evaluators in the healthy eyes group (p = 0.006) but not in the affected eyes group (p = 0.083). Bacterial presence was detected by both evaluators, and the findings did not differ statistically from the bacteriology results (p = 0.05). Significant variations were noted in the differential cell count; the mean count of the superficial epithelial cells and goblet cells of the healthy eyes group (p < 0.05) and that of the basal/intermediate cells and neutrophils of the affected eyes (p < 0.05) showed significant differences.

**Conclusion:**

The evaluator’s experience significantly affected the differential cell count in both the healthy and affected eyes groups. Neutrophil degeneration was not observed by the less experienced evaluator, whereas bacteria were detected equally well by both the evaluators.

## Introduction

Ophthalmological examination of varying complexity is performed by veterinarians in general practice and veterinary ophthalmologists [1, 2]. Microscopic examination is relatively easy to perform, but the usefulness of this test is affected by the sample quality, such as the cellularity, cell preservation, and the evaluator’s experience [3, 4]. The commonly used sampling methods with a cotton swab, cytobrush, or spatula can disrupt the normal cellular architecture to a certain degree [4]. Sampling techniques that do not disrupt the cellular architecture much have been proposed, such as impression cytology; however, as they are time, labor, and equipment intensive, they are yet to be introduced in daily clinical practice [3, 4].

Although several studies have assessed the interobserver agreement for ocular cytology samples [3, 4], our approach was different. We included both healthy and affected eye samples and aimed to assess those parameters that were most likely to be considered important by general practitioners in daily clinical practice. There are several studies comparing different testing or sampling methods as well as interobserver differences [3, 4], but a general practitioner is not likely to implement a lengthy procedure or invest in a single purpose equipment. Our study was specifically aimed to use only the equipment and sampling methods that would be used in a general practice/first opinion clinic.

This study aimed to evaluate whether the cytology parameters of cellularity, cell preservation, inadequate sample rate, differential cell counts, neutrophil morphology assessment, and detection of bacteria differed between healthy and affected canine eyes when evaluated by variably experienced personnel.

## Materials and Methods

### Ethical approval and Informed consent

This study was approved by the Ethics Committee of the Latvia University of Life Sciences and Technologies (LLU_Dzaep2022-1-1). Written informed consent was obtained from the dog owners before including their dogs in this study.

### Study period and location

The study was conducted from September 2020 to January 2022 at the Small Animal Clinic and Clinical Institute of the Faculty of Veterinary Medicine, Latvia University of Life Sciences and Technologies, Latvia.

### Animals and sampling

All animals examined were privately owned and were outpatients at the LLU veterinary clinic. We included 34 dogs of both sexes (14 females and 20 males) and various breeds, aged 8 months–13 years. All the dogs underwent routine clinical and ophthalmological examinations.

The ophthalmological examination included direct ophthalmoscopy (Keeler Practitioner, Windsor, UK), monocular ophthalmoscopy using PanOptic Ophthalmoscope (Welch Allyn, Romford, UK), slit-lamp biomicroscopy (Kowa SL15, Nagoya, Aichi, Japan), and rebound tonometry (TonoVet^®^, Tiolat Ltd., Finland). To ensure uniformity in the results, the ophthalmological examination was conducted by the same veterinary ophthalmologist (3^rd^ author).

Based on the results of the ophthalmological examination, the dogs were divided into the healthy eyes group (clinically and ophthalmologically healthy dogs) and the affected eyes group (dogs diagnosed with conjunctivitis and/or keratoconjunctivitis). The dogs included in the affected eyes group had to have at least three of the following symptoms: Conjunctival edema, hyperemia, discharge, blepharospasm, and itchiness. Routine health checkup results did not influence the categorization of the dogs into the healthy eyes group or affected eyes group.

After clinical examination, cytology samples were obtained from both eyes. Approximately 30 s after administering one drop of topical proxymetacaine hydrochloride (5 mg/mL; Alcaine, Alcon-Couvreur, Belgium) with a sterile cotton swab, the bulbar conjunctiva was gently swabbed. The sample was transferred to a glass slide by gentle rolling of the swab. The slide was air-dried and stained using Dip Quik (JorVet, USA). Evaluator I (2^nd^ author) mostly had theoretical experience in ocular cytology that comprised reviewing of books, pictures, atlases, and online materials [2, 5–8]. Evaluator II (1^st^ author) had undergone clinical pathology residency training and had 16 years of practical experience.

Microscopically, the following criteria were assessed:

- Cellularity was assessed at 100× in 10 alternating fields of view across the whole slide. The number of intact and recognizable cells in the monolayer was counted in each view field, and the mean cell number was calculated (score 3, mean number of cells >71; score 1, mean number of cells <35) (Table-1).
- Cell preservation was assessed at 1000× in 10 representative view fields (score 3, >71% of the cells were intact in the 10 view fields; score 1, <35% of the cells were intact).
- An inadequate sample rate was calculated for the healthy and affected eyes groups. The sample was considered inadequate if cellularity was <35% and cell preservation rate was 1.
- Differential cell count (superficial epithelial cells, basal/intermediate epithelial cells, goblet cells, neutrophils, lymphocytes, and macrophages) was performed at 1000× in the representative view fields.
- Presence of mucus and melanin granules was observed during the differential cell count.
- Cell morphology assessment with emphasis on degenerate neutrophils was performed simultaneously with the differential cell count.
- Bacterial presence or absence was noted.
  Bacteriological evaluations were performed for all the samples. Both evaluators were blinded to the clinical examinations and bacteriology results.

**Table 1:** Semi-quantitative scoring system to evaluate the cellularity, cell preservation, and mucus content of the samples.

Score	1	2	3
Percentage of cells	<35	36–70	>71
Cellularity	Scant	Adequate	Abundant
Cell preservation	Fair	Good	Excellent
Mucus content	Not present	Moderate	Abundant

### Statistical analysis

Agreement between the two evaluators for the semi-quantitative parameters was assessed using the weighted Cohen’s kappa method [3, 9]. Statistical analyses were performed using Statistical Product and Service Solutions (SPSS, version 12.0.0, SPSS Inc., Chicago, IL, USA); p < 0.05 was considered statistically significant.

## Results

In this study, 20 dogs (40 healthy eye cytology samples) were included in the healthy eyes group and 14 dogs in the affected eyes group (28 affected eye cytology samples). The inadequate sample rate in the healthy eyes group was 1/40 by Evaluator I and 9/40 by Evaluator II (p = 0.006841), while that in the affected eyes group was 5/28 and 1/28, respectively (p = 0.083953).

Regarding cell preservation in the healthy eyes group, both evaluators showed agreement in 25 cases: Excellent, 22/25; good, 2/25; and fair, 1/25. In contrast, the evaluators showed agreement in 15 cases in the affected eyes group: Excellent, 8/15; good, 6/15; and fair, 1/15.

The overall agreement between the evaluators regarding the cellularity parameters of the sample was better in the affected eyes group than in the healthy eyes group (Table-2).

**Table 2:** Agreement between two variably experienced evaluators regarding the quality parameters of the cytology samples.

Parameters	Cohen’s kappa value	Level of agreement
Cellularity in healthy eyes	0.000	Similar to that expected by chance
Cell preservation in healthy eyes	0.213	Fair
Mucus content in healthy eyes	0.214	Fair
Cellularity in affected eyes	0.571	Moderate
Cell preservation in affected eyes	0.253	Fair
Mucus content in affected eyes	0.563	Moderate

Statistically significant differences were noted in the differential cell count (Table-3). There were significant differences in the mean percentages of the superficial epithelial and goblet cells in the healthy eyes groups and those of the basal/intermediate cells and neutrophils in the affected eyes group (p < 0.01 for both groups). Evaluator I did not observe degenerate neutrophils in the healthy eyes or affected eyes, whereas Evaluator II found degenerate neutrophils in two affected eye samples, wherein bacteria were not detected on microscopy but the bacteriology result was positive.

**Table 3:** Parameters evaluated by two variably experienced evaluators.

Parameters	Evaluator I	Evaluator II	p-value
Inadequate cellularity in healthy eyes	1/40	9/40	**<0.01**
Inadequate cellularity in affected eyes	5/28	1/28	0.19
Inadequate cell preservation in healthy eyes	1/40	9/40	**<0.01**
Inadequate cell preservation in affected eyes	1/28	5/28	**0.03**
Inadequate sample rate in healthy eyes	1/40	9/40	**<0.05**
Inadequate sample rate in affected eyes	5/28	1/28	0.08
Superficial epithelial cells in healthy eyes (mean)	31.35	10.43	**<0.01**
Basal/intermediate cells in healthy eyes (mean)	61.25	64.77	0.28
Goblet cells in healthy eyes (mean)	0.27	8.05	**<0.01**
Neutrophils in healthy eyes (mean)	0.13	0.30	0.16
Superficial epithelial cells in affected eyes (mean)	10	14.85	0.07
Basal/intermediate cells in affected eyes (mean)	76.10	50.25	**<0.01**
Goblet cells in affected eyes (mean)	2.53	6.46	0.09
Neutrophils in affected eyes (mean)	11.25	16.21	**<0.01**
Bacteria in affected eyes	0/12	5/12	0.05

Bold values indicate statistical significance.

Bacteria were not observed by either evaluator in the healthy eyes group. Evaluator I observed bacteria in three affected eye samples. Bacteria were not observed in either of the samples by Evaluator II, and none of those samples showed positive bacteriology results. The agreement between Evaluator II and the bacteriology results in the affected eye samples was 5/12, but the difference between the cytological findings and bacteriology results was not statistically different for either of the evaluators (p = 0.05).

## Discussion

A swab of the conjunctiva produces poorly cellular, moderately preserved, and well-distributed cytology smears [2]. The agreement between the two evaluators in our study on these sample parameters was better in the affected eyes group than in the healthy eyes group. This difference could be explained by the presence of healthy cells in larger clusters or sheets [10] compared to the high proportion of individual inflammatory cells in the affected eyes. Cytology textbooks and materials available online contain few pictures of normal cells and mostly focus on the pathologies that can be observed. Based on our study, we can state that lack of visual representation of normal cytology can affect less experienced evaluators’ performance [5]. We recommend for all evaluators to familiarize themselves with the normal cytological features of conjunctiva.

We observed a significant difference in the sample adequacy assessment in the healthy eyes group. This finding corroborates the findings of other studies on various tissues; the inadequate sample rate was high in the benign and/or healthy tissue cytology samples [11, 12]. The inadequate sample rates were lower in the affected eyes group than in healthy eyes group, which could be attributed to the high number of intact individual cells that could be detected by both evaluators. Ocular cytology samples are rather small and may be unevenly distributed, presenting both thick and thin areas; hence, evaluation may be challenging [8]. Therefore, we can conclude that differentiation among the epithelial cells is more challenging.

An important finding of our study was the difference in the observation of degenerate neutrophils. It is possible that less experienced evaluators could mistake degenerated neutrophils as lysed cells. Degeneration is characterized by an enlarged, swollen, light staining nucleus, the nuclear membrane may be fuzzy, cytoplasmic border is intact, and cytoplasm may be vacuolated and contain intracytoplasmic bacteria; however, degeneration can be observed even when the number of bacteria is too low to detect cytologically [5]. Degeneration signifies the possibility of sepsis and bacteriology should be performed [7].

In this study, there was no difference in the evaluators’ abilities to detect bacteria at the significance level of statistical difference set by us. No bacteria were detected in the healthy eyes, but bacteriology results were positive in 34/40 of these samples. The healthy eye samples testing positive for bacteriology had no clinical or cytological evidence of inflammatory disease; hence, these bacteria are probably the normal microflora of the eyes [8]. In the affected eyes, bacteria were observed along with high neutrophil counts and in correlation with the clinical signs. Evaluator I observed cocci bacteria in three cases, none of which showed positive bacteriology results; Evaluator II did not detect any bacteria. The literature suggests that bacteria are mostly mistaken for melanin granules, stain precipitates, or ultrasonography gel [13]. Considerable experience is needed to differentiate them based on color and shape as well as to correlate these findings with other clinical and microscopic observations.

Significant differences in the differential cell count were observed in healthy and affected eyes groups. Evaluator I underestimated the neutrophil percentage in the affected eyes group compared to that in the healthy eyes group, which could be attributed to the degeneration and altered morphology of these cells in the former. Degeneration was not observed in any of the samples by Evaluator I, which supports this possibility. The increased number of neutrophils is an important finding that guides clinical and treatment decisions [10].

Significant differences in the number of goblet cells in healthy dog eyes are an important finding. Dry eye syndrome is commonly observed in dogs, and the number of goblet cells, along with the results of Schirmer’s tear test and other quick tests significantly contributes to the diagnosis [10].

The fact that we opted not to evaluate the cytoplasmic and nuclear details could be a limitation of this study; however, the evaluation of these cellular details is important for the diagnosis of neoplasia, which was not the aim of this study. Several authors have evaluated cellular details such as the cytoplasm, nuclear features, and chromatin [3, 4]. We chose to evaluate the differences in the differential cell count between the evaluators because this is the most vital parameter for general practitioners in clinical practice.

## Conclusion

Considering the specific characteristics of the ocular cytology samples, including different cell types and uneven distribution of the sample, the evaluators’ experience significantly affected the sample adequacy assessment, differential cell count, and cellular morphology assessment. These factors significantly impact on the clinical decisions in both healthy and sick dogs. We cannot rule out the possibility of bacterial overgrowth or post-sampling contamination. Extended analysis of the bacteriology results will be performed and published separately.

## Authors’ Contributions

LK: Conceptualized, designed, and planned the study and supervised the analysis, and corrected the manuscript. LK: Ophthalmological examination and sampling. AT and IB: Microscopy, evaluation, statistical analysis, and writing of the manuscript. All authors have read and approved the final manuscript.
